# Spatial Variability in COVID-19 Mortality

**DOI:** 10.3390/ijerph18115892

**Published:** 2021-05-31

**Authors:** Brian H. Bossak, Catrina A. Turk

**Affiliations:** Department of Health and Human Performance, College of Charleston, Charleston, SC 29424, USA; turkca@g.cofc.edu

**Keywords:** COVID-19, vitamin D, omega-3, spatial variability, SARS-CoV-2, diet, case-fatality rate, CFR

## Abstract

COVID-19 emerged as a global pandemic in the spring of 2020. Since that time, the disease has resulted in approximately 150 million cases and 3 million deaths worldwide. However, there is significant spatial variation in the rate of mortality from COVID-19. Here, we briefly explore spatial variations in COVID-19 mortality by country groupings and propose possible explanations for the differences observed. Specifically, we find that there is a statistically significant difference in COVID-19 mortality between countries grouped into categories based on (1) developed, primarily western diets and healthcare systems; (2) “Scandinavian” countries with advanced healthcare systems and generally anti-inflammatory diets, and (3) developing countries. We do not infer causality but believe that the observed associations provide hypotheses for future research investigations. Moreover, our results add further evidence to support additional exploration of vitamin D exposure/status and COVID-19 mortality.

## 1. Introduction

COVID-19 (SARS-CoV-2 virus) emerged in late 2019 and rapidly became the next significant human pandemic. As of March 19th, 2021, global COVID-19 incidence was 122,489,228 cases, with 2,705,366 deaths [1]. Considerable spatial variation in the incidence of COVID-19 has been associated with a variety of factors, such as country preparedness, access to healthcare, implementation of public health interventions, and the population’s underlying health status [2,3,4,5]. In regard to individual health status, an increased risk of developing a respiratory illness is associated with chronic diseases, obesity, and vitamin deficiency [6]. COVID-19 virulence has been associated with individuals characterized as being in high-risk groups, such as the elderly, minority groups, and individuals that suffer from chronic diseases; however, many of these same individuals are susceptible to vitamin D deficiency, which may influence the severity of COVID-19 and other respiratory illnesses, particularly those with a viral origin. 

Vitamins play a fundamental role in the function of the immune system and can influence immune response [6]. Vitamin D is a steroid hormone that is associated with the production of antimicrobial peptides and the expression of genes involved in the destruction of pathogens; as such, it has an important role in the induction of the innate immune response which could provide protection against influenza-like illnesses [7]. Mechanistically, it can influence the presence of receptors on the cell surface which recognize pathogenic proteins and regulate genes that are used for defenses against viruses [8]. It is also involved in protection against acute lung injury and reduces lung permeability [6]. Studies suggest that high mortality or prevalence of respiratory illnesses are associated with the cool and dry climate that occurs during the winter months [9]. However, there is reason to believe that vitamin D deficiency may serve as a confounder in the pathway between exposure to a viral pathogen and subsequent infection (and virulence of such) that could explain the seasonality and/or severity of respiratory epidemics [10]. We sought to examine whether this particular vitamin can be correlated to the prevalence of COVID-19 by analysis of COVID-19 mortality in developing countries versus developed countries, with level of economic development serving as an assumed proxy for vitamin D status (e.g., less developed country = greater average outdoor exposure = less general vitamin D deficiency).

## 2. Materials and Methods

A total of 52 countries were selected for the analysis. Data regarding COVID-19 mortalities per 1 M population for these countries were retrieved from the Worldometers website, which contains disease data that is collated daily from official government reports [1]. The data was sorted, and countries were assigned to categories with the highest COVID-19 mortality rates per 1 M population and the countries with the lowest COVID-19 mortality rates per 1 M population. The countries found to have higher mortality rates were mostly developed countries (Developed) while the countries with the lowest mortality rate tended to be developing countries (Developing). A *T*-test (95% confidence) was employed to determine if there was a statistically significant difference in the mean mortality rate per 1 M population between the 20 countries with the most COVID-19 deaths per 1 M population and the 20 countries with the lowest number of deaths per 1 M. 

**Figure 1:**
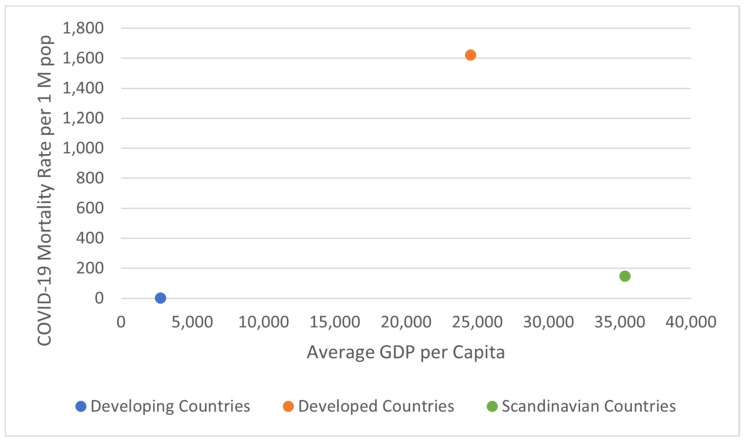
Scatter plot of the average COVID-19 death rate per 1 M population versus average GDP of each group of countries, as of 30 March 2021. A KW test on COVID-19 mortality per 1 M population showed a statistically significant difference for these three groupings of countries (*p* < 0.01).

**Figure 2:**
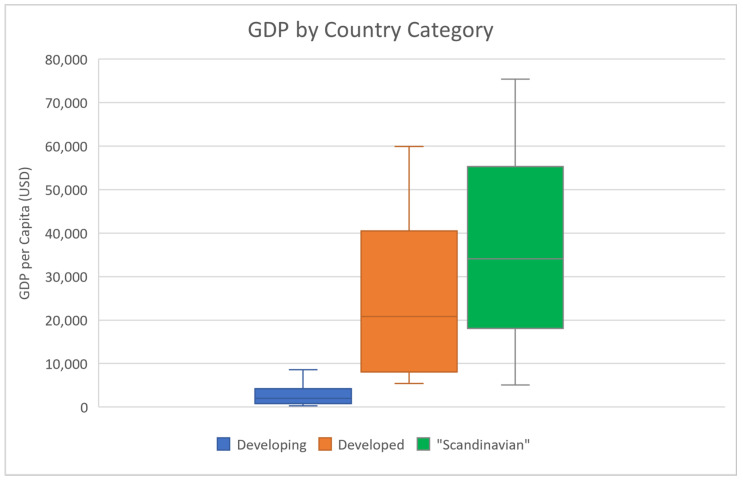
Box plot of GDP per capita for each grouping of countries (USD).

Another factor that could be influencing the spatial heterogeneity of COVID-19 severity is lifestyle, specifically diet. Interestingly, many highly developed Scandinavian countries show a much lower severity of COVID-19 (Table 1; data from www.worldometers.info/coronavirus/ (accessed on 30 March 2021)) as represented by deaths per 1 M population (Figure 1; data from the Worldometers website). The categorization of “Scandinavian” countries as a grouping of countries here more broadly refers to medically advanced nations with potentially similar dietary exposures to anti-inflammatory foods and higher seafood/fish consumption than other country groupings, rather than solely based on geographic location in Scandinavia. Moreover, these “Scandinavian” countries possess the highest GDP per capita of the three groups (Table 2 and Figure 2; data from www.worldometers.info/gdp/gdp-per-capita (accessed on 30 March 2021)). We tested the distribution of the COVID-19 mortality rates in each country categorization for normality using the Kolmogorov-Smirnov Test for Normality. While both the Developing and “Scandinavian” country groupings were normally distributed, the mortality data for the Developed country category was non-normally distributed. As a result, we utilized the non-parametric Kruskal-Wallis (KW) test to determine if there was a statistically significant difference between COVID-19 mortality in “Scandinavian” countries compared to the 20 countries with the highest mortality rate per 1 M and the 18 countries with the lowest mortality rate per 1 M (two countries from the initial analysis of the 20 countries with the lowest COVID-19 mortality per 1 M population were re-classified into the “Scandinavian” group for further analysis: New Zealand and Singapore). 

## 3. Results

Countries that were found to have the highest death rates per 1 M population and the countries that were found to have the lowest death rates per 1 M population for COVID-19 were tested for statistical significance. A *T*-test demonstrated that there is a statistically significant difference between COVID-19 mortality in developed countries and COVID-19 mortality in developing countries + two of the “Scandinavian” countries as described above (*p* < 0.001). Moreover, this significant difference was found to be directional; there is a much higher rate of COVID-19 mortality per 1 M population in developed countries versus developing countries. The results of the Kruskal-Wallis (KW) test indicated a statistically significant difference between the mortality rates of the three groupings of countries (*H* = 11.7815, *p*-value = 0.00276). Thus, the results suggest that there is spatial variability in the reported COVID-19 severity between developing, developed, and Scandinavian-type countries as defined here. 

There are many possible explanations for the observed statistical difference in mortality rates. This vast difference in severity could be associated with other mechanisms such as better healthcare or higher rates of testing. However, there is one factor that is different between “Scandinavian” countries and other western countries that are exhibiting drastically higher mortality from COVID-19. Many “Scandinavian” countries tend to incorporate an abundance of seafood into their diets which could account for some of the difference in COVID-19 severity. Seafood contains vitamin D; therefore, this surplus of vitamin D incorporated into their diet could demonstrate the difference of severity between developed countries. Moreover, many types of seafood are high in levels of omega-3 fatty acids, which contain anti-inflammatory properties. The developed countries that have a high death rate for COVID-19 per 1 M population tend to have more saturated fats, sugar, and red meat in their diets. Therefore, there could be multiple factors related to diet, lifestyle, and vitamin D synthesis that could be associated with the severity of COVID-19 infection distinguished by national identities, perhaps partly explaining why there is such a drastic difference in COVID-19 mortality rates between developed countries, developing countries, and “Scandinavian” countries.

## 4. Discussion

Our analysis demonstrates that there is a statistically significant difference in COVID-19 severity between countries (and country groupings) and across space. One possible explanation is that the significant difference in COVID-19 severity may be associated with vitamin D exposure or lack thereof, therefore correlating with vitamin D deficiency. Vitamin D status is largely associated with exposure to sunlight which is significantly reduced in the winter months and could contribute to the increased prevalence in respiratory illnesses during this time [8]. In most developing countries, lifestyle tends to be targeted towards outdoor activities which could reduce prevalence of vitamin D deficiency. In developed countries, sun exposure may generally be more limited because a greater portion of the lifespan is being spent indoors (office workers, institutionalized populations, retail, and others). This could be one factor associated with the increasing prevalence of vitamin D deficiency in developed countries which would have a corresponding effect on immune response: therefore, possibly increasing severe cases of respiratory illnesses for a given population size. For example, this association has been demonstrated in the elderly population, which also tends to be one of the most susceptible populations to COVID-19. Many elderly individuals have vitamin D deficiency due to reduced amount of vitamin D in their diet, decreased skin synthesis of vitamin D, and limited time outdoors [11].

However, these differences are more evident when COVID-19 severity is stratified by economic development factors (Figure 1) rather than simply location and become more pronounced when lifestyle and dietary considerations are explored. While we cannot infer a causal association between lifestyle factors, such as time spent outdoors or exposure to sunlight, these are distinctive parameters within and between each grouping of countries and have been introduced in association with health and disease in prior studies [12,13]. Moreover, different countries responded to the pandemic in a spectrum of actions, which may partially explain the observed spatial variation in mortality rates; for example, Italy was one of the countries with a significant outbreak of COVID-19 early in the pandemic, albeit in a region of the country (Po Valley) distinct from the rest of the state [14]. Within and between countries, ambient environmental conditions may also have influenced aggregate COVID-19 mortality, such as genetics, climate, and humidity [15]. 

In terms of potential weaknesses or limitations to our approach, we recognize that country-level reporting of cases and deaths from COVID-19 could influence these results. For example, underreporting or delayed reporting of cases or deaths in countries indicating low COVID-19 morbidity/mortality could bias any correlative statistical associations. It is possible that associations between GDP per capita and COVID-19 mortality may also be spurious or random. However, many of the countries that have reporting low COVID-19 mortality per 1 M population to this point in the pandemic (“Scandinavian”) have advanced healthcare systems with no strong reason to underreport. Moreover, we utilize mortality data which may be somewhat less likely to suffer from underreporting bias than COVID-19 incidence data. 

## 5. Conclusions

There was a significant correlation between the number of COVID-19 deaths per 1 M population and the level of development within and between countries. Surprisingly, the rate of mortality per 1 M population was higher in western developed countries with respect to developing nations and/or countries with the highest development of healthcare provision (termed “Scandinavian”). There may be numerous factors that could explain these observed differences, including some that may be associated with the lifestyle and societal norms in specific countries (e.g., diet and nutrition, physical activity, genetics, environment, sunlight exposure, or other factors). The factors considered here need further exploration; however, if additional evidence that vitamin D deficiency be a factor in the variance of COVID-19 mortality is observed, this could suggest vitamin D supplementation as a possible public health intervention in the future. The interaction(s) between vitamin D and its role in general immune status (as well as specific associations with COVID-19 severity) merit further studies. Early research results on these purported associations support further exploration of this potential factor on disease outcomes and virulence [16,17,18,19]. Moreover, associations between COVID-19 mortality and diet/lifestyle factors, such as nutritional status or consumption of seafood, warrant further exploration.

## Figures and Tables

**Table 1:** The COVID-19 mortality rate per 1 M population for the 20 countries with the highest and lowest rates, and “Scandinavian” countries (Singapore and New Zealand moved to “Scandinavian” here).

Developed Countries	Rate of Death per 1 M Pop	Developing Countries	Rate of Death per 1 M Pop	“Scandinavian” Countries	Rate of Death per 1 M Pop
Gibraltar	2672	Burundi	0.2	Japan	61
San Marino	2119	Tanzania	0.3	Denmark	405
Belgium	1883	Vietnam	0.4	South Korea	31
Slovenia	1816	Taiwan	0.4	Iceland	85
Czechia	1792	Mongolia	0.6	Norway	114
UK	1770	Thailand	1	Estonia	143
Italy	1585	Papua New Guinea	1	Finland	134
Portugal	1568	Bhutan	1	Uruguay	171
USA	1535	Eritrea	2	Cuba	28
Bosnia and Herzegovina	1524	Fiji	2	Cypress	190
Montenegro	1512	Western Sahara	2	Australia	35
Hungary	1483	China	3	Jamaica	139
North Macedonia	1470	Benin	6	Singapore	5
Spain	1435	Uganda	7	New Zealand	5
Bulgaria	1423	Ivory Coast	7		
Liechtenstein	1414	Guinea	7		
Mexico	1385	Burkina Faso	7		
Andorra	1383	Niger	7		
Peru	1349				
Croatia	1328				
Average	1622		3		149

**Table 2:** The GDP per capita for the countries with the highest and lowest death rates per 1 M population and “Scandinavian” countries.

Developed Countries	GDP per Capita	Developing Countries	GDP per Capita	“Scandinavian” Countries	GDP per Capita
Gibraltar	N/A	Burundi	293	Japan	38,214
San Marino	48,495	Tanzania	975	Denmark	57,545
Belgium	43,325	Vietnam	2366	South Korea	24,490
Slovenia	N/A	Taiwan	N/A	Iceland	29,958
Czechia	20,291	Mongolia	3672	Norway	75,428
UK	39,532	Thailand	6579	Estonia	20,170
Italy	32,038	Papua New Guinea	2434	Finland	45,778
Portugal	21,316	Bhutan	3391	Uruguay	16,341
USA	59,939	Eritrea	N/A	Cuba	8541
Bosnia and Herzegovina	5387	Fiji	5768	Cypress	18,695
Montenegro	7720	Western Sahara	N/A	Australia	54,831
Hungary	14,364	China	8612	Jamaica	5061
North Macedonia	5418	Benin	827	Singapore	56,746
Spain	28,175	Uganda	631	New Zealand	43,415
Bulgaria	8197	Ivory Coast	1529		
Liechtenstein	N/A	Guinea	868		
Mexico	9224	Burkina Faso	642		
Andorra	39,128	Niger	376		
Peru	6723				
Croatia	13,200				
Average	23,675		2756		35,372

## Data Availability

https://www.worldometers.info/coronavirus/ (accessed on 30 March 2021) and www.worldometers.info/gdp/gdp-per-capita (accessed on 30 March 2021).
